# German ambulatory care physicians' perspectives on clinical guidelines – a national survey

**DOI:** 10.1186/1471-2296-7-47

**Published:** 2006-07-20

**Authors:** Martin Butzlaff, Daniela Kempkens, Melanie Schnee, Wilfried E Dieterle, Jan Böcken, Monika A Rieger

**Affiliations:** 1 Competence Center for General Medicine and Outpatients' Health Care, Faculty of Medicine, University Witten/Herdecke, Alfred-Herrhausen-Str. 50, D 58448 Witten, Germany; 2 Bertelsmann Stiftung, Gütersloh, Germany, Carl-Bertelsmann-Str. 256, PO Box 103, D 33311 Gütersloh, Germany; 3 University Hospital for Psychiatry and Psychosomatic Medicine, Department of Psychosomatic and Psychotherapeutic Medicine, University Hospital of Freiburg, Hauptstr. 8, D 79104 Freiburg, Germany

## Abstract

**Background:**

There has been little systematic research about the extent to which German physicians accept or reject the concept and practice of

a) clinical practice guidelines (CPG) and

b) evidence based medicine (EBM)

The aim of this study was to investigate German office-based physicians' perspective on CPGs and EBM and their application in medical practice.

**Methods:**

Structured national telephone survey of ambulatory care physicians, four thematic blocks with 21 questions (5 point Likert scale). 511 office-based general practitioners and specialists. Main outcome measures were the application of Clinical Practice Guidelines in daily practice, preference for sources of guidelines and degree of knowledge and acceptance of EBM. In the data analysis Pearson's correlation coefficient was used for explorative analysis of correlations. The comparison of groups was performed by Student's t-test. Chi^2 ^test was used to investigate distribution of two or more categorical variables.

**Results:**

Of the total study population 55.3% of physicians reported already using guidelines in the treatment of patients. Physicians in group practices (GrP) as well as general practitioners (GP) agreed significantly more with the usefulness of guidelines as a basis for patient care than doctors in single practices (SP) or specialists (S) (Student's t-test mean GP 2.57, S 2.84, p < 0.01; mean GrP 2.55, SP 2.80, p < 0.05). 33.1% of the participants demonstrated a strong rejection to the application of guidelines in patient care. Acceptance of guidelines from a governmental institution was substantially lower than from physician networks or medical societies (36.2% vs. 53.4% vs. 62.0%). 73.8% of doctors interpret EBM as a combination of scientific research and individual medical knowledge; 80% regard EBM as the best basis for patient care.

**Conclusion:**

Despite a majority of physicians accepting and applying CPGs a large group remains that is critical and opposed to the utilization of CPGs in daily practice and to the concept of EBM in general. Doctors in single practice and specialists appear to be more critical than physicians in group practices and GPs. Future research is needed to evaluate the willingness to acquire necessary knowledge and skills for the promotion and routine application of CPGs.

## Background

Clinical decisions of everyday patient care are based on scientific medical knowledge as well as personal experience of the practicing physician. However, a large gap remains between what we know and what we practice [1]. Physicians have to cope with a rapidly growing amount of new medical knowledge. Apart from relevant and high-quality publications, they are confronted with an increasing amount of irrelevant and useless information. The ability to differentiate between these is becoming a key competence for the individual practitioner [2].

The concept of evidence based medicine (EBM) as "the conscientious, explicit, and judicious use of current best evidence" and "integration of individual clinical expertise" [3] offers a theoretical framework to combine scientifically generated knowledge and personal experience. Since the term "evidence based medicine" was first coined in the early 1990s, the theoretical concept led to a rapidly broadening scientific discourse, initially concentrated in the Anglo-American academic world. Within a few years of EBM's rapid dissemination and application in scientific journals, universities, and medical societies, critical voices also started to appear [4-7]. Today, evidence based Clinical Practice Guidelines (CPG) are seen as a cornerstone and as important tools for the implementation and dissemination of the concept of EBM [8,9]. Thus, CPGs are promoted as key instruments for health care improvement in most industrialized countries.

With a delay of a few years the same development took place in Germany: In 1998 the first EBM-specific scientific society was founded and the topic of EBM started to appear in calls for research proposals by major scientific institutions [10]. Today EBM is promoted by all important governmental institutions of the German healthcare system [11,12].

The reaction to EBM outside the academic and political community was quite different. Many hospital and office-based physicians were skeptical: The concept of EBM and CPGs as one of the main instruments of EBM-implementation into daily patient care were regarded as threats to a high degree of professional autonomy in medical decision-making [13].

There has been little systematic research about the extent to which German physicians accept or reject the practical application of CPGs and the concept of EBM – 15 years after its scientific introduction.

To gain a better understanding of the current status and future prospects for implementation of CPGs in German health care reform initiatives, the following questions were developed as part of a representative survey of 500 office-based general practitioners and specialists:

1. Are CPGs accepted and used in daily medical practice?

2. Which institutions, as authors and editors of CPGs, are trusted most?

3. Does acceptance and implementation differ in various areas of ambulatory care or in different medical specialties? Is it possible to identify potential problems with implementation and deficits which then need to be addressed in the future?

4. To which extent is the concept of EBM known to German office-based physicians?

## Methods

The data of this study was taken from the project "Healthcare Monitor" of the Bertelsmann Stiftung. Since 2001, office-based physicians as well as patients and health-insured citizens are questioned on a regular basis about the topic of ambulatory care by this health-survey. In November 2003, primary care physicians (general practitioners, internists, pediatricians, obstetricians/gynecologists) and specialists were interviewed by telephone through a polling institute using a standardized questionnaire. According to previous experience by the polling institute, sample size was determined to be 500 physicians. The sample was a disproportionally stratified quota sample of 250 primary care physicians and 250 specialists. Stratification characteristics were frequency and distribution of physicians according to specialty and state, based on the information of the German Medical Association and the National Association of Statutory Health Insurance Physicians. Age or numbers of patients treated per three-month period were not included as characteristics as there is only regional, non-comparable data available. Gender was not included because experience shows that distribution in the quota matches the national distribution. According to the stratification characteristics the interviewers recruited physicians by phone during their consultation hours until 250 primary care physicians and 250 specialists had completed the interview. Exact response rate was not recorded by the polling institute. The estimated response rate was between 14 and 20%. In addition, on average 3.5 telephone contacts with the interviewee were necessary to realize the interview. Interviewed doctors received a nominal fee as a compensation for their time and effort.

As Germany has no gatekeeping system for GPs the physicians included in the primary care group of the Healthcare Monitor are often the first point of contact for patients in the healthcare system. However, in this study we applied an international widely accepted definition of primary care physicians, and therefore chose a different grouping for analysis as follows: This paper assigned general practitioners, internists working as general practitioners, and pediatricians to the group of primary care, if doing house calls. Obstetricians/gynecologists and internists working as specialists and all other specialists were assigned to the specialist group.

The "Healthcare Monitor" – physician sample 2003 entails questions about the following topics: "medical decision-making" (21 questions), "continuing medical education" (37), "the informed patient" (20) as well as questions about socio-demographic factors. This paper focuses on the topic of "professional autonomy in medical decision-making", which is subdivided into four thematic blocks:

1. Best treatment options of the physician for the patients (block 1, 4 items)

2. Meaning of evidence based medicine for physicians (block 2, 6 items)

3. Current utilization and application of guidelines (block 3, 5 items)

4. Preference of important characteristics of guidelines (e.g. origin) (block 4, 6 items).

The interviewed physicians were asked to judge statements in these four thematic blocks on a five point Likert-scale (1 = "completely agree" to 5 = "strongly disagree"). During the descriptive analysis categories were summarized into three groups:

- agree = "completely agree" + "mostly agree"

- disagree = "mostly disagree" + "strongly disagree"

- undecided = "to some extent"

After a general descriptive analysis of the data, we performed three main analyses. First, we analyzed potentially influential factors through explorative correlation between socio-demographic factors and attitudes. Following this, Student's t-test was carried out (between two groups of special interest, namely general practitioners versus specialists and physicians in a group practice versus those working in single practice. Finally, we analyzed the response pattern of physicians who were especially reluctant to use guidelines. Principal components analysis (PCA) was applied as the most common form of factor analysis to detect the structure in the relationships within these patterns. PCA was used to identify the main dimension within the response patterns in thematic block one. Based on the main dimension of this thematic block two distinct groups were formed thus differentiating according to the physicians' attitudes towards guidelines. Ethics approval was not required for this study.

The data analysis was conducted with commercial standard software (SPSS 11.5, SPSS Inc., Chicago, IL). Pearson's correlation coefficient was used for the explorative analysis of correlations. The comparison of groups was performed by Student's t-test. Chi^2 ^test was used to investigate the distribution of two or more categorical variables. In the following sections standard deviation is indicated by the sign ±.

## Results

### Description of study population

Of 511 interviewed office-based physicians, 368 were male and 143 female (28%). The average age of the study population was 51.3 years; 26.8% of the physicians were 45 years old or younger, in the group of the 46 to 54 year-olds there were 36.4% and 36.8% were at least 55 years of age.

According to the chosen definition the study population consisted of 212 (41.5%) primary care physicians and 299 (58.5%) specialists. These two groups differed significantly (p < 0.05) in their percentage of women: 33.0% in primary care and 24.4% in specialties.

Of the interviewed physicians 70.1% worked as the only doctor in their practice, 28.5% worked in group practice and 1.4% in polyclinics. Physicians who worked in a group practice were older than physicians in single practice (53.9 ± 8.6 vs. 51.0 ± 8.3 years, p = 0.001). The percentage of single or group practices did not differ between primary care physicians and specialists.

There was a considerable correspondence with the available data about office-based physicians in Germany: the study population was slightly older, the percentage of women slightly lower and the number of doctors working in single practice marginally higher than in the comparative group (table 1) [8].

**Table 1:** Comparison study population with German national data

	**Study population**	**Office-based physicians**	**p-value**
Average age (years)	51.3	50.5	<0.05*
Women (%)	28.0	32.0	0.05^+^
Working in single practice (%)	70.1	68.6	0.48^+^

* Student's t-test

+ Chi^2^

On average the practice was 13.5 years old and the age upon starting to work as an office-based physician was 37.9 years. Primary care practices were 1.7 years older than specialist practices (14.5 vs. 12.8 years, p < 0.05). The average age at start of work as an office-based physician was similar in both groups (37.3 vs. 38.3 years, no significant difference).

Specialists treated on average more patients per three-month period than primary care physicians (primary care physicians 1000–11200 patients vs. specialist 1200–1400 patients, p < 0.001).

### Frequency of answers in the four thematic blocks

#### What is the best option for physicians to treat the patients?

**Figure 1:**
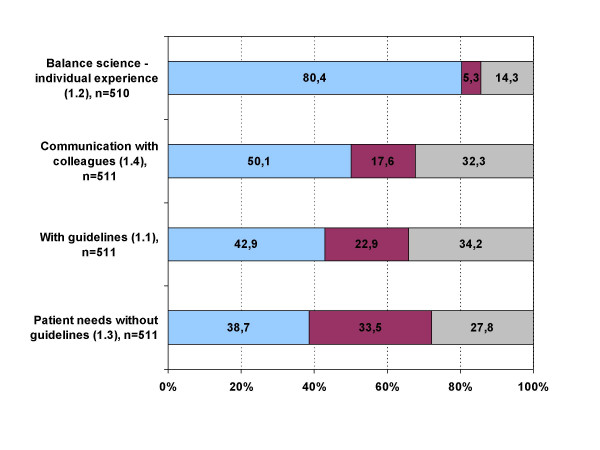
**Thematic block one: Best basis for patient treatment**. blue: agree; red: disagree; grey: undecided; Block 1: "The physician can best treat the patient... 1.1 ..on the basis of scientific knowledge in form of guidelines." 1.2 ..in the balance of scientific recommendations, individual needs and current possibilities." 1.3 ..without guidelines and with the knowledge of individual needs and patient's possibilities." 1.4 ..on the basis of continuous communication with colleagues."

80.4% of interviewed physicians felt that patients were best treated "in the balance of scientific recommendations, individual needs and current possibilities." Half of the study population agreed with the statement that patients can be optimally treated "on the basis of continuous communication with colleagues". On the other hand 38.8% of physicians thought that patients are best treated "without guidelines and with the knowledge of individual needs and patient's possibilities" (figure 1).

#### To which extent are guidelines already applied in daily care of patients?

**Figure 2:**
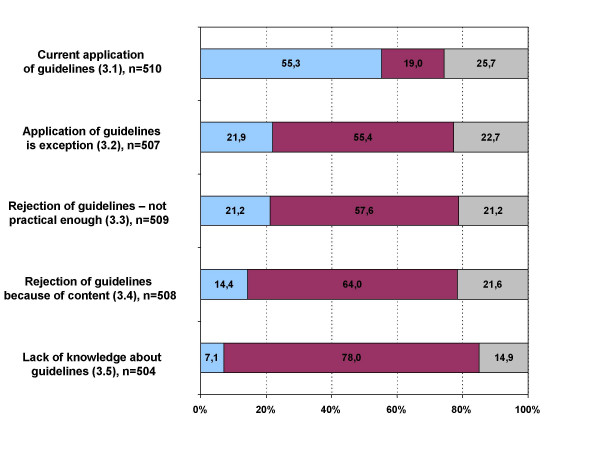
**Thematic block three: Application of guidelines in clinical practice**. blue: agree; red: disagree; grey: undecided; Block 3: "In the care of my patients I... 3.1 ..already work with guidelines." 3.2 ..use guidelines only as an exception." 3.3 ..reject guidelines, because they are not practical enough." 3.4 ..reject guidelines, because I do not support their content." 3.5 ..so far do not work with guidelines, because I do not know enough about them."

In 55.3% of the cases physicians stated that they already used guidelines in the care of their patients, 21.9% applied guidelines only as an exception and only 7.1% agreed with the statement that limited knowledge about guidelines prevents their clinical application. As a reason for rejecting guidelines, 21.2% said that guidelines were not practical enough, 14.4% that they did not support their content (figure 2).

#### Which sources of guidelines are preferred?

**Figure 3:**
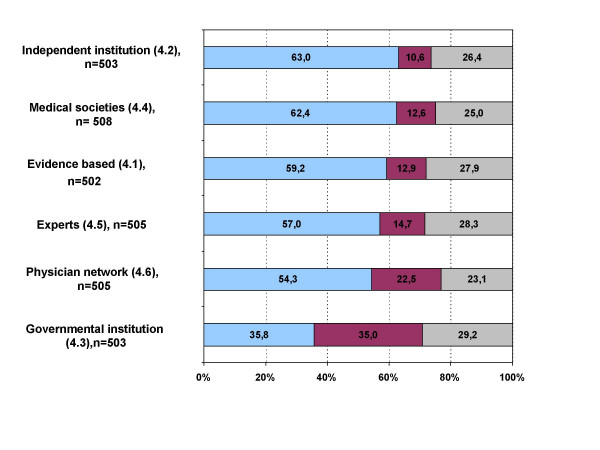
**Thematic block four: Origin and characteristics of guidelines**. blue: agree; red: disagree; grey: undecided; Block 4: "I would work with guidelines which... 4.1 ..are evidence based." 4.2 ..are evidence based and were developed by an independent institution (i.e. university)." 4.3 ..are evidence based and were developed by a governmental institution for quality assurance." 4.4 ..were developed by medical societies." 4.5 ..were developed by experts." 4.6 ..were developed by a network of physicians, which I belong to."

The rate of acceptance – each about 60% – was relatively high for guidelines which were evidence based (59.2%), were evidence based and developed by an independent institution or university (63.0%), were developed by medical societies (62.4%) or experts (57.8%). Guidelines which originated from a physicians' network to which the individual physician belonged would be used by 54.3% of the interviewed physicians. Only 35.8% of the study population would use guidelines which are evidence based and developed by a governmental institution for quality assurance (figure 3).

#### What is the subjective meaning of "evidence based medicine" for physicians?

**Figure 4:**
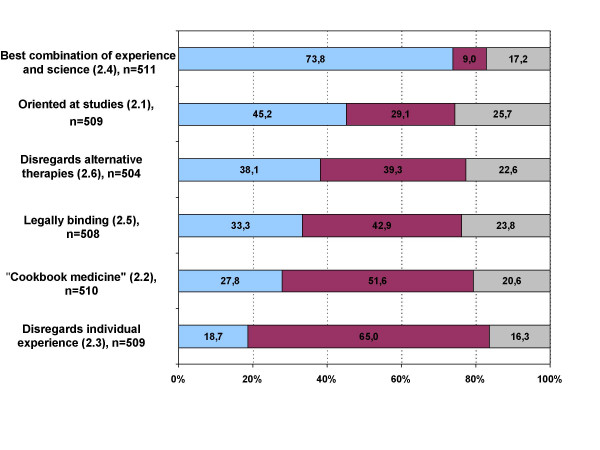
**Thematic block two: Meaning of evidence based medicine**. blue: agree; red: disagree; grey: undecided; Block 2: "Evidence based medicine means to me... 2.1 ..medicine, which is exclusively orientated towards scientific studies." 2.2 ..medicine, which treats patients with the same diagnosis in exactly the same way (*'cookbook medicine'*)." 2.3 ..medicine, which ignores the physician's individual medical experience." 2.4 ..to combine my individual experience with the best available evidence from systematic research." 2.5 ..to work with lawfully binding guidelines, which impair my professional autonomy in medical decision-making." 2.6 ..medicine, which ignores alternative therapies."

For the majority of physicians (73.8%) evidence based medicine signifies the combination of "individual experience with the best available evidence from systematic research." 45.2% of doctors understood EBM as medicine, "which is exclusively oriented towards scientific studies". EBM was viewed by 38.1% as medicine, "which ignores alternative therapies", 33.3% defined it as work "with legally binding guidelines" which "impair my professional autonomy in medical decision-making". The statement that EBM is medicine which "treats patients with the same diagnosis in exactly the same way (cookbook-medicine)" was agreed to by 27.8%, 18.7% thought that this concept "ignores the physician's individual medical experience" (figure 4).

### Influence of baseline characteristics on response pattern in the four thematic blocks

The explorative screening found no influence of gender on response patterns in the four thematic blocks. There were correlations between the factors age, age of practice, numbers of patients in practice as well as patients treated multiple times per three-month period with various items in the subdivisions. The significant results are summarized in table  2. The mean is calculated from the 5-point Likert-scale.

**Table 2:** Influence of baseline characteristics on response pattern (data from Healthcare Monitor 2003)

**Thematic block**	**Factor of influence**				**Total**
**Block 1: „The physician can best treat the patient...**					

1.1 ..on the basis of scientific knowledge – in form of guidelines."	**Age (years)**	**≤45**	**46 to 54**	**≥55**	
	Degree of agreement n	134	182	184	500
	%	41.8	35.2	50.5	42.6
	**Age of practice (years)**	**≤10**	**11–20**	**≥21**	
	Degree of agreement n	167	243	93	503
	%	39.5	42.0	49.5	42.5

**Block 3: „In the care of my patients I...**					

3.2 ..use guidelines only as an exception."	**No of staff/practice**	**1–3**	**4–5**	**≥6**	
	Degree of agreement n	151	191	160	502
	%	28.5	20.9	16.3	21.7
	**Number of patients in practice/three-month period**	**<1000**	**1000–1400**	**≥1400**	
	Degree of agreement n	150	129	165	444
	%	26.7	25.6	15.8	22.0
3.3 ..reject guidelines, because they are not practical enough."	**Age of practice (years)**	**≤10**	**11–20**	**≥21**	
	Degree of agreement n	166	242	93	500
	%	16.3	22.3	28.0	21.4
3.5 ..so far do not work with guidelines, because I do not know enough about them."	**Age of practice (years)**	**≤10**	**11–20**	**≥21**	
	Degree of agreement n	165	238	93	496
	%	2.4	8.8	9.7	6.9
	**Patients seen multiple time/3-month period**	**<30%**	**30–50%**	**≥50%**	
	Degree of agreement n	138	174	185	497
	%	10.9	7.5	4.3	7.2

**Block 4: "I would work with guidelines which...**					

4.4..were developed by medical societies."	**Age of practice (years)**	**≤10**	**11–20**	**≥21**	
	Degree of agreement n	167	241	92	500
	%	64.7	62.2	57.6	62.2
4.6..were developed by a network of physicians, which I belong to."	**Age (years)**	**≤45**	**46 to 54**	**≥55**	
	Degree of agreement n	133	181	178	492
	%	65.4	56.3	44.4	54.5
	**Age of practice (years)**	**≤10**	**11–20**	**≥21**	
	Degree of agreement n	166	237	92	495
	%	66.3	48.9	44.6	53.9

### Differences between single and group practices

**Figure 5:**
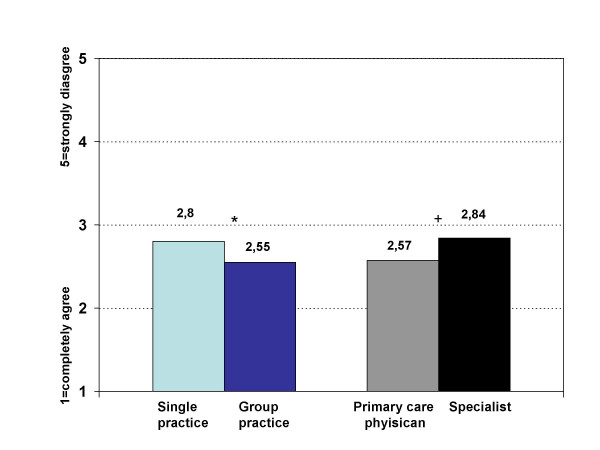
**Guidelines as best basis for patient treatment – type of practice and primary care physicians vs. specialists**. * p < 0.05 + p < 0.01

Student's t-test of the groups single and group practice found significant differences for two questions. Both groups were indifferent towards the statement that the physician can best treat the patient on the basis of scientific knowledge in form of guidelines, but single practices agreed less than group practices (mean single physician practice 2.80, group practice 2.55, p < 0.05) (figure 5).

Both groups disagreed with the statement that guidelines were rejected on ground of their content, but physicians in group practices disagreed more strongly (mean single physician practice 3.30, group practice 3.77, p < 0.01).

### Differences between primary care physicians and specialists

Three significant differences were found between the groups of primary care physicians and specialists. Both groups were indifferent towards the statement that the physician can best treat the patient on the basis of scientific knowledge in form of guidelines, but specialists agreed less than primary care physicians (mean primary care physician 2.57, specialist 2.84, p < 0.01) (figure 5).

Specialists agreed more than primary care physicians with the statement that patients can best be treated on the basis of continuous communication with colleagues (mean primary care physician 2.72, specialist 2.53, p < 0.01).

Although both groups would rather not use guidelines which were developed by a governmental institution, specialists showed slightly more dislike than primary care physicians (mean primary care physician 2.90, specialist 3.12, p = 0.062).

### Significance of guidelines in treatment of patients – formation of groups according to certain characteristics

**Figure 6:**
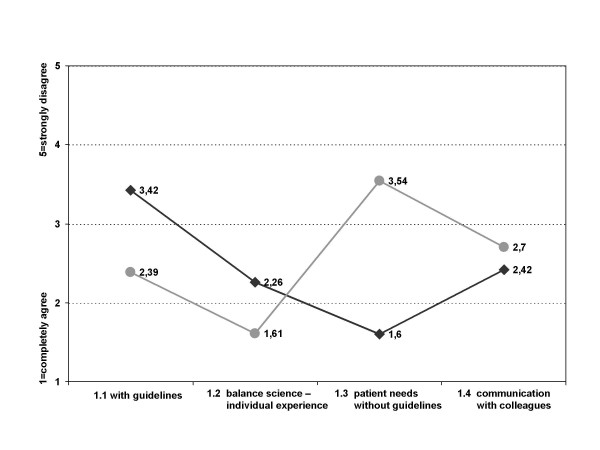
**Response pattern of Agr-Gr and Dis-Gr in thematic block one (mean)**. black: Agr-Gr; grey: Dis-Gr; Block 1: "The physician can best treat the patient... 1.1 ..on the basis of scientific knowledge in form of guidelines." 1.2 ..in the balance of scientific recommendations, individual needs and current possibilities." 1.3 ..without guidelines and with the knowledge of individual needs and patient's possibilities." 1.4 ..on the basis of continuous communication with colleagues."

A principal component analysis (PCA) of the response patterns in thematic block one (basis for treatment of patients) revealed that the attitude towards the statement "the physician can best treat the patient without guidelines and with the knowledge of individual needs and patient's possibilities" (question 1.3) was juxtaposed to the other statements. The physicians' opinion about the significance of guidelines for the treatment of patients was clearly reflected by the attitude towards this central marker-item. Based on these results, two groups of physicians were distinguished and compared in a comprehensive analysis that included all items. The first group (Agr-Gr, n = 169, 33.1%) clearly agreed with the statement "the physician can best treat the patient without guidelines and with the knowledge of individual needs and patient's possibilities", whereas the second group (Dis-Gr, n = 342, 66.9%) clearly disagreed with the statement. The Dis-Gr agreed with the central statement for understanding EBM, "the physician can best treat the patient in the balance of scientific recommendations, individual needs and current possibilities" (question 1.2) more strongly than the Agr-Gr (figure 6).

The two groups did not differ in socio-demographic or structural data. In comparison of the groups, significant differences were shown consistently in the three other thematic blocks (question 2, 3, 4).

The judgment of statements regarding current application of guidelines (thematic block 3) consistently differed between these two groups. The Agr-Gr said that they used guidelines significantly less in daily practice [mean total group (Tot.) 2.47, Dis-Gr 2.25, Agr-Gr 2.92, p < 0.001 (1 = completely agree, 3 = undecided, 5 = strongly disagree)] and only as an exception compared to the Dis-Gr (mean Tot. 3.50, Dis-Gr 3.75, Agr-Gr 2.97, p < 0.001). Reasons for rejection of guidelines in the index group were neither lack of practice orientation, issues of content, or lack of knowledge about guidelines.

**Figure 7:**
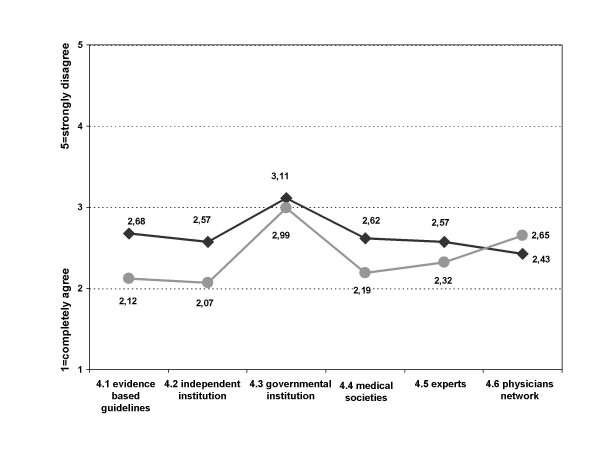
**Response pattern of Agr-Gr and Dis-Gr in thematic block four (mean)**. black: Agr-Gr; grey: Dis-Gr; Block 4: "I would work with guidelines which... 4.1 ..are evidence based." 4.2 ..are evidence based and were developed by an independent institution (i.e. university)." 4.3 ..are evidence based and were developed by a governmental institution for quality assurance." 4.4 ..were developed by medical societies." 4.5 ..were developed by experts." 4.6 ..were developed by a network of physicians, which I belong to."

Further differences between the two groups were noted in respect to preference of certain guideline characteristics (thematic block 4). In comparison to the rest of the study population the Agr-Gr would use guidelines less that were evidence based (question 4.1) (mean Tot. 2.30, Dis-Gr 2.12, Agr-Gr 2.68, p < 0.001), evidence-based and developed by an independent institution (mean Tot. 2.23, Dis-Gr 2.07 Agr-Gr 2.54, p < 0.001), developed by medical societies (mean Tot. 2.33, Dis-Gr 2.19, Agr-Gr 2.62, p < 0.001) or experts (mean Tot. 2.40, Dis-Gr 2.32, Agr-Gr 2.57, p < 0.05). Both groups were rather undecided about guidelines that were evidence based and developed by a governmental institution (mean Tot. 3.03, Dis-Gr 2.99, Agr-Gr 3.11, p = 0.28). Guidelines developed by a network of physicians to which they belonged themselves were most agreeable to the Agr-Gr but showed no difference to the Dis-Gr (mean Tot. 2.57, Dis-Gr 2.65, Agr-Gr 2.43, p = 0.6) (figure 7).

Evaluating the importance of EBM for physicians, the Agr-Gr agreed more with the statement that EBM "ignores alternative therapies", but overall physicians disagreed slightly with this statement (Tot. 3.86, Dis-Gr 3.97, Agr-Gr 3.65, p < 0.05).

The Dis-Gr rated the statement that EBM means "to combine individual experience with the best available evidence from systematic research" higher than the Agr-Gr; overall the rate of agreement to this item was high (mean Tot. 1.98, Dis-Gr 1.90, Agr-Gr 2.15, p < 0.01).

## Discussion

This representative survey provides insight into the utilization of clinical practice guidelines and the knowledge and acceptance of evidence based medicine by German office-based physicians. Several results are relevant for the next steps of health care reform in Germany:

Clinical practice guidelines appear to have reached "the real world". More than half of all physicians in this survey stated that they already used guidelines in practice. However, a substantial fraction of German office base physicians remains skeptical, using CPGs only as an exception or rejecting CPGs due to their content or due to deficits in practicability. Although most physicians favour CPGs which are evidence based, it cannot be estimated to which extent CPGs, which were already being used in practice, are following an evidence based concept.

The relatively strong agreement of doctors to guidelines developed by a university or medical society was remarkable (app. 60%), especially in the light of only one third of the study population who would rely on guidelines issued by a governmental institution for quality assurance. This "trust-gap" needs special attention. As there is currently intense promotion for the implementation of CPGs through the political institutions in Germany, all well intended initiatives may fail, or at least not reach their full potential, if they are predominantly seen as instruments of control by a majority of practicing doctors.

Whereas the general subjective agreement to evidence based medicine was high, this pattern clearly lessened when physicians were asked about regular use of guidelines in daily practice: half of the physicians interviewed already used guidelines in the treatment of patients. However, one in five doctors either rejected guidelines because of their inadequacies for daily practice or because of lack of support for their content. There is a clear gap, well described in many studies about the implementation of guidelines [14-16], between theoretically agreeing to the concept of EBM and practical application of guidelines which are based on scientific evidence. Thus, a major research question of the past decade will need continuous and intensified attention: which strategies for guideline implementation are useful for specific environments and different groups of physicians [8,17]?

Overall, there is a positive attitude among German office-based physicians towards evidence based medicine: three out of four doctors interpret EBM according to the original definition [3] as a combination of scientific research and individual medical knowledge; four out of five physicians regard this as the best basis for patient care. A similar attitude among general practitioners was already shown five years ago in the UK and Australia [18-20]. A regional study in 2004 demonstrated similar results for a group of office-based physicians in the North of Germany [21].

At the same time one in four doctors was in agreement with the view of EBM as "*cookbook-medicine*", as well as best patient care without the aid of guidelines. This clearly shows that a significant group of German doctors associate EBM with a loss of professional autonomy.

For many physicians systematic research and individual clinical expertise continue to be contradicting methods in daily medical practice, even though both elements are fundamental to the definition of EBM and many examples for clinical practicability exist [22].

A more differentiated picture of German physicians' perspective on CPGs and EBM was achieved by looking at single and group practices as well as primary care physicians and specialists. Physicians in group practices as well as primary care practitioners agreed much more with the usefulness of guidelines as a basis for patient care than doctors in single practices or specialists. This gives rise to the questions concerning which subgroups of physicians are especially willing or unwilling to practice evidence based patient care and how the latter group could be motivated to make the transition to evidence based care.

A further analysis was carried out, comparing a group of physicians (Agr-Gr) who demonstrated a strong rejection to the application of guidelines in patient care to colleagues (Dis-Gr) affirming the use of guidelines. This skeptical attitude towards guidelines of a third of the study population is consistently mirrored in the answers of other major questions: the correct definition of EBM is less recognized, guidelines are not regularly used in clinical practice, and evidence based guidelines are less trusted. This heterogeneous subgroup could not be characterized further by socio-demographic factors compared to the other physicians; no differences were found for age, gender, type or size of practice.

In 2004 two major reforms of the German health care system were implemented which have the potential to influence the attitudes towards evidence based medicine and guidelines of doctors significantly: For the first time continuing medical education has become mandatory for all office-based physicians. The German medical association recommends evidence based content and the use of clinical guidelines.

An initiative by the German government led to the formation of a national institute for quality and cost effectiveness in health care. One of its responsibilities is to present current medical knowledge and critically appraise evidence based guidelines.

## Conclusion

Despite a majority of physicians accepting and applying CPGs a large group remains that is critical and opposed to the utilization of CPGs in daily practice and to the concept of EBM in general. Doctors in single practice and specialists appear to be more critical than physicians in group practices and GPs. Future research is needed to evaluate the willingness to acquire necessary knowledge and skills for the promotion and routine application of CPGs.

## Limitations

The following limitations of this study need to be outlined: The stratified random sample of physicians was interviewed by telephone. Selection bias through physicians more willing to respond to a telephone survey may have influenced the results. However, the available comparable data shows the great concordance to the national mean with respect to type of practice, and gender, the age of the sample investigated being slightly higher in the study population. In addition, the distribution of age and gender is similar to a regional study of 574 office-based physicians in the North of Germany [21]. This survey is an explorative study, no primary and secondary outcomes were determined beforehand. Significant differences between different groups of doctors, as shown by statistical analysis, help to generate hypothesis. Social desirability is another main factor which has to be taken into account in every survey. The subjective pattern of response, i.e. in the subjective report on guideline utilization, cannot be validated with the present data. Studies of validity have shown that actual behavior and skills can differ greatly.

## Future research questions

The results of this study lead to further research questions that could be important to the continuous implementation of evidence based medicine in German medical practice:

1. Is the awareness of EBM accompanied by willingness to apply evidence based knowledge in the form of clinical practice guidelines?

2. How can mandatory continuing medical education (since 2004) be effectively utilized to achieve reasonable dissemination and acceptance of evidence based guidelines in Germany?

3. Are there ways to overcome the distrust of a large number of German physicians of governmental health care institutions in the implementation of EBM and what are effective means of communication and instruments to motivate doctors with a skeptical opinion of EBM?

## Competing interests

The author(s) declare that they have no competing interests.

## Authors' contributions

MB and MR conceived the idea for the paper. WD, MR, MS, JB and DK carried out the data analysis. MB and DK wrote the original draft of the paper, all authors contributed to further drafting. MB is guarantor.

## Pre-publication history

The pre-publication history for this paper can be accessed here:


